# A Flexible Pulse Generator Based on a Field Programmable Gate Array Architecture for Functional Electrical Stimulation

**DOI:** 10.3389/fnins.2021.702781

**Published:** 2022-01-21

**Authors:** Jorge A. Mercado-Gutierrez, Ricardo Dominguez, Ignacio Hernandez-Popo, Jimena Quinzaños-Fresnedo, Arturo Vera-Hernandez, Lorenzo Leija-Salas, Josefina Gutierrez-Martinez

**Affiliations:** ^1^Departamento de Ingeniería Eléctrica, Sección Bioelectrónica, Centro de Investigación y de Estudios Avanzados del Instituto Politécnico Nacional, Mexico City, Mexico; ^2^División de Investigación en Ingeniería Médica, Instituto Nacional de Rehabilitación Luis Guillermo Ibarra Ibarra, Mexico City, Mexico; ^3^Departamento de Ingeniería Eléctrica, Universidad Autónoma Metropolitana — Iztapalapa, Mexico City, Mexico; ^4^CONACYT — Instituto Nacional de Rehabilitación Luis Guillermo Ibarra Ibarra, Mexico City, Mexico; ^5^División de Rehabilitación Neurológica, Instituto Nacional de Rehabilitación Luis Guillermo Ibarra Ibarra, Mexico City, Mexico

**Keywords:** field programmable gate array, functional electric stimulation, bioelectronics, frequency modulation, neurorehabilitation, upper limb, neuroprosthesis, muscle fatigue

## Abstract

Non-invasive Functional Electrical Stimulation (FES) is a technique applied for motor rehabilitation of patients with central nervous system injury. This technique requires programmable multichannel systems to configure the stimulation parameters (amplitude, frequency, and pulse width). Most FES systems are based on microcontrollers with fixed architecture; this limits the control of the parameters and the scaling to multiple channels. Although field programmable gate arrays (FPGA) have been used in FES systems as alternative to microcontrollers, most of them focus on signal acquisition, processing, or communication functions, or are for invasive stimulation. A few FES systems report using FPGAs for parameter configuration and pulse generation in non-invasive FES. However, generally they limit the value of the frequency or amplitude parameters to enable multichannel operation. This restricts free selection of parameters and implementation of modulation patterns, previously reported to delay FES-induced muscle fatigue. To overcome those limitations, this paper presents a proof-of-concept (technology readiness level three-TRL 3) regarding the technical feasibility and potential use of an FPGA-based pulse generator for non-invasive FES applications (PG-nFES). The main aims were: (1) the development of a flexible pulse generator for FES applications and (2) to perform a proof-of-concept of the system, comprising: electrical characterization of the stimulation parameters, and verification of its potential for upper limb FES applications. Biphasic stimulation pulses with high linearity (*r*^2^ > 0.9998) and repeatability (>0.81) were achieved by combining the PG-nFES with a current-controlled output stage. Average percentage error in the characterizations was under 3% for amplitude (1–48 mA) and pulse width (20–400 μs), and 0% for frequency (10–150 Hz). A six-channel version of the PG-nFES was implemented to demonstrate the scalability feature. The independence of parameters was tested with three patterns of co-modulation of two parameters. Moreover, two complete FES channels were implemented and the claimed features of the PG-nFES were verified by performing upper limb functional movements involving the hand and the arm. Finally, the system enabled implementation of a stimulation pattern with co-modulation of frequency and pulse width, applied successfully for efficient elbow during repetitions of a functional movement.

## Introduction

In recent years, several rehabilitation strategies have been used to address the motor sequelae of brain and spinal cord injuries. One example is the Functional Electrical Stimulation (FES) technique, used in neurological rehabilitation programs to improve motor function and manual dexterity (Popovic et al., 2001). This technique is based on the application of low intensity, biphasic electrical impulses that produce action potentials in peripheral nerves and generate muscle contractions (Eraifej et al., 2017). The literature reports the use of FES systems as an effective approach to induce functional rehabilitation of the upper limb (Quandt and Hummel, 2014; Osuagwu et al., 2016). The design of electrical stimulation systems has evolved to fulfill the growing needs of FES applications, from dedicated customed-designed analog control circuitry (Vodovnik et al., 1967), to configurable, high-speed multifunction digital systems (Kutlu et al., 2017) which employ highly integrated programmable devices.

Most FES systems reported in the research literature (Chung et al., 2004; Popovic et al., 2005; Jovičić et al., 2012; Luzio de Melo et al., 2015; Venugopalan et al., 2015) are based on microcontrollers. These devices manage the configuration and timing of the stimulation parameters: amplitude, frequency and pulse-width (Stewart et al., 2016). However, microcontrollers have a fixed and low number of timers and of pulse-width modulation blocks, and they execute one instruction at a time. Those features limit the design of FES systems (Stewart et al., 2016) regarding the number of stimulation channels and their flexibility for independent control of stimulation parameters, two important features in FES applications (Kesar et al., 2008; Grimm and Gharabaghi, 2016). In the other hand, there are design strategies for FES systems that use programmable devices known as field programmable gate arrays (FPGAs). The main advantage of using an FPGA for this end is the large number of logic blocks arrays and interconnections, which are programmable and reconfigurable. FPGAs allow the implementation and almost simultaneous execution of several complex digital functions. Additionally, they require a small board space, are energy efficient and have a low non-recurring engineering cost. Therefore, FPGAs have enabled the design of customized digital electronic systems for several applications, such as digital control, communication interfaces, signals and image processing, computer algorithms, machine learning, and big data (Ruiz-Rosero et al., 2019).

From the 1990s, FPGAs have been used to develop FES systems. They have been applied for Peripheral Nervous System (PNS) stimulation, like bladder (Sawan et al., 1996; Arabi and Sawan, 1999), respiration (Zbrzeski et al., 2016; Castelli et al., 2017), and visual prostheses (Wong et al., 2005). Also, they have been used for Central Nervous System (CNS) stimulation, including intracortical (Sugiura et al., 2016) and deep brain stimulation (Karami et al., 2016). Moreover, FPGAs have been applied to implement various functions in FES systems, such as data communication (Thanasoulis et al., 2012), signal acquisition (Hsueh et al., 2015), signal processing (Ying et al., 2007; Hsueh et al., 2015; Ranganathan et al., 2019), and control (Ambroise et al., 2013; Karami et al., 2016; Shahdoost et al., 2017).

A key function in FES systems is the generation of stimulation pulses and the control of their main parameters: pulse duration, amplitude, and frequency. These parameters largely determine the activation of the target biological structures, and they need to be adapted for each stimulation channel, according to the context of the FES application. Thus, FPGAs can be useful to implement the required functions for configuration and management of stimulation parameters in an FES system. FES systems using FPGAs for the generation and control of stimulation pulses have been reported since the 1990s (Sawan et al., 1996) up to very recently (Ranganathan et al., 2019). However, most of them were designed for invasive applications, with pulse amplitude values in the range of hundreds of μA (Amerijckx et al., 1998; Castelli et al., 2017) to a few mA (Sawan et al., 1996; Simpson and Ghovanloo, 2007). In contrast, non-invasive FES applications require larger electrodes (up to tens of cm^2^) placed over the skin (transcutaneous), which increase the pulse amplitude requirements: from a few to tens of mA.

There are few reports of transcutaneous (non-invasive) electrical stimulation systems utilizing FPGAs (Benzaba et al., 2008; Chen et al., 2009; Annetta et al., 2019). Benzaba et al. (2008) presented the design of a Transcutaneous Electrical Nerve Stimulation (TENS) system, using an FPGA for the control of parameters of monophasic stimulation pulses. However, they presented only computer simulations and preliminary tests of the system, which did not demonstrate the actual functionality of the system. A year later, Chen et al. (2009) developed a FES system for eyelid reanimation in patients with facial paralysis. They used an FPGA for signal processing of EMG signals to detect eye blinks and send activation commands to a programmable 8-channel digitally controlled current stimulator, implemented on an Application Specific Integrated Circuit (ASIC). That system generated biphasic stimulation pulses with maximum amplitude of 6 mA.

More recently, Annetta et al. (2019) reported a multichannel FES system based on two FPGA circuits. This system controls an array of small stimulation electrodes over the forearm, which produce a variety of movements of the fingers, wrist and forearm. However, they used a monophasic pulse shape to simplify the design, acknowledging the convenience of biphasic stimulation pulses for safer, long-term interventions. Moreover, their wired communication interface constrained its portability and limited the stimulation pulse rate to a maximum of 50 pulses per second (divided in the number of active channels at a time). Also, the maximum stimulation amplitude was 20 μA, which would prevent their use with standard surface stimulation electrodes (10–50 cm^2^), used in FES clinical practice.

Previous FPGA-based FES systems have not taken full advantage of their programmable core device, to allow high flexibility in the configuration of stimulation parameters. On the contrary, they often limit some parameters to fixed (amplitude or frequency) or dependent values (frequency) among different channels. Furthermore, this restrains the development and use of electrical stimulation patterns comprising modulation of parameters, a relevant approach for minimizing muscle fatigue during the use of FES (Ibitoye et al., 2016). Early muscle fatigue is a great limiting factor for the use of FES interventions aimed to motor rehabilitation of people with neurological conditions (Thrasher et al., 2005), reducing the stimulation time and the potential therapeutic effects. Several approaches have been proposed in the past for modulation of one or more parameters, in order to decrease reduce or delay fatigue and to enhance the performance of FES applications (Ibitoye et al., 2016).

Commercial electrical stimulation systems often allow modulation of a single stimulation parameter at a time: pulse width or amplitude. However, previous works have suggested that co-modulation of two (Gregory et al., 2007; Chou et al., 2008; Downey et al., 2011) or more (Nekoukar, 2021) stimulation parameters can help to reduce early FES-induced muscle fatigue. However, some of those works required the implementation of custom hardware solutions to obtain the desired stimulation patterns (Downey et al., 2011; Nekoukar, 2021). Furthermore, these solutions are aimed to single-channel stimulation of the quadriceps muscle (Gregory et al., 2007; Kesar et al., 2008; Downey et al., 2011). Thus, they are not optimal for clinical research in FES applications, for example, of the upper limb.

Taking into consideration the previous ideas, there is a niche for the design of configurable electrical stimulation systems using an FPGA to handle the pulse generation function, with flexible control of stimulation parameters suitable for non-invasive FES applications, and a scalable architecture to multiple stimulation channels. The main aims of this work are: (1) to develop the prototype of a pulse generator for non-invasive FES based on an FPGA (PG-nFES) and (2) to perform a proof-of-concept of the system, comprising the electrical test bench characterization of the stimulation parameters of the full range stimulation parameters and the verification of the system’s potential for transcutaneous FES applications.

The design of the PG-nFES takes advantage of FPGA features to integrate, in a single device, a flexible digital pulse generator for biphasic stimulation, the device control logic, and a serial communication interface for configuration of the stimulation parameters. Moreover, the architecture of the PG-nFES is designed to be scalable, a highly desirable feature for the design of multichannel electrical stimulation systems for FES applications.

## Materials and Methods

### Development of the Pulse Generator for Non-invasive FES Applications

The development of the pulse generator for non-invasive FES (PG-nFES) is divided in three stages: first, the identification of critical design specifications, second, the design and implementation of the system, and third, the characterization and proof-of-concept of the system of the system. These stages are described below.

#### Design Specifications

The main technical specifications for the design of the PG-nFES are summarized in Table 1.

**TABLE 1:** Required specifications and features of the FPGA-based architecture of the PG-nFES.

Design specification	Description
Pulse shape	Rectangular, biphasic
Range of stimulation parameters	Frequency: 1–150 Hz. Pulse width: 20–400 μs. Amplitude: 1–50 mA. Burst time: 1–4 s active (9–6 s rest) Additional mode: Continuous stimulation
Control of parameters	Independent, parameter-wise, and channel-wise.
Scalability	Allows extension to a higher number of stimulation channels
Safety features	Battery-powered, isolated power supply, emergency stop
Interfaces	Buttons for selection of parameters and functions, Inputs for triggering stimulation bursts. Serial interface for communication with controlling devices

#### Field Programmable Gate Arrays Features and Programming

To implement the PG-nFES, a Spartan ^®^-6 XC6SLX9-2TQG144I FPGA (Xilinx ^®^ Inc., San Jose CA, United States) was used, containing 715 Configurable Logic Blocks, 9152 gates and 576 kb of RAM. This device is embedded in the Mojo v3, an FPGA prototyping board with a main clock frequency of 50 MHz, 84 digital input/output pins, and an ATMEGA32U4 microcontroller for programming the FPGA, analog signal acquisition and digital interfacing.

The FPGA code was written in the ISE Design Suite v 14.7 (Xilinx ^®^ Inc., San Jose CA, United States) software, using the Verilog hardware description language. Once the binary programming file was generated by ISE, the design was loaded to the FPGA *via* an USB port of the PC, using the Mojo Loader v1.3.0 application.

### Design and Implementation

This subsection describes the design of the system, including the overall architecture and signal flow inside the FPGA, and the external circuitry for amplitude control. Also, it describes the settings of the switches for the selection of the operation modes and the active channel. Finally, the inner workings of the pulse generation blocks are explained.

#### System Architecture

**FIGURE 1:**
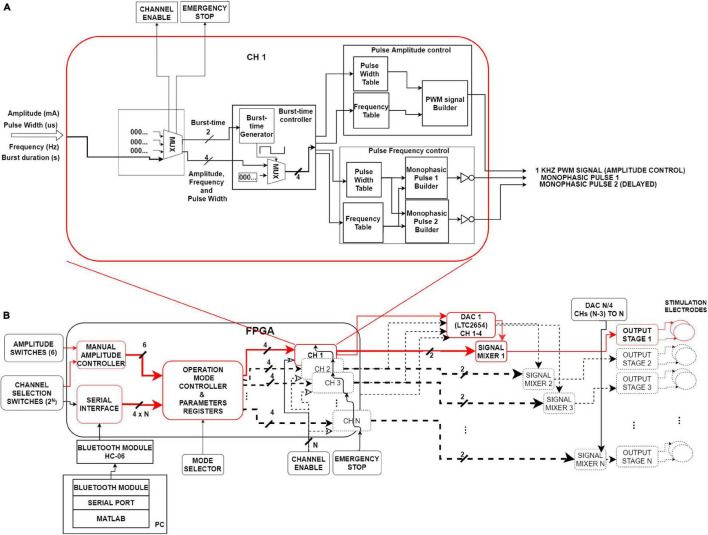
System’s architecture. **(A)** Internal diagram of a single channel (CH1) of the PG-nFES. This block forms two time-shifted monophasic pulses (frequency and pulse width), and one PMW signal (amplitude). **(B)** Left. Digital interfaces and logic blocks for management and storing of stimulation options and parameters, and the main PG-nFES blocks (CH 1 to CH N). Right. Analog blocks transform the three FPGA outputs to a pair of monophasic pulses with voltage controlled-amplitude, which are combined in the output stage into a single biphasic, current-controlled stimulation pulses. Red blocks and arrows illustrate the implementation of a single FES channel. Blocks with black dotted lines sketch the scaling capability of the architecture from one to N channels.

Figure 1 shows the block diagram of the FPGA-based architecture of the PG-nFES, including:

– Digital interfaces and configuration modules of stimulation options and parameters (Figure 1B, left).
– Modules for building digital pulses and bursts (Figure 1A).
– External circuits to the FPGA, that convert the pairs of monophasic voltage pulses, to biphasic current-controlled pulses (Figure 1B, right).

Below is given a general description of the signal flow within the PG-nFES, with reference to the Figure 1. The main task of the FPGA-based modules is to generate two time-shifted, monophasic pulses with the desired frequency, pulse-width, and burst-time parameters, and one PWM signal containing the amplitude information. Once those signals leave the FPGA, the PWM signal enters the DAC, which in turn generates a DC voltage output signal that modulates the amplitude of the pair of monophasic pulses in the signal mixers. After these conditioning stages, the two monophasic pulses enter the output stage (Leyva et al., 2018), where they are converted to a single, current-controlled, biphasic stimulation pulse with the desired parameters.

Figure 1B highlights (in red) the blocks and signals of the PG-nFES which were built and tested in this work (one stimulation channel). The blocks in black dotted lines illustrate the elements of this architecture that need to be replicated to scale up the system from one to N channels. The next subsections detail the architecture and main functions of the PG-FES.

#### Operation of the Pulse Generator

The PG-nFES includes several digital inputs and a serial interface, which are used for setting stimulation parameters and operation modes of the system. The functions of these modules are described in Table 2. The following steps describe the procedure to initialize and configure the stimulation parameters of the PG-nFES. The modules involved in this procedure are those illustrated in Figure 1B, unless otherwise noted.

**TABLE 2:** Functions of digital inputs and the serial interface of the PG-nFES.

	Input/function name (size)	Function description
Digital inputs (Switches)	*Channel enable* (N bits, 1 p/channel)	Switches to enable/disable the stimulation output of each channel
	*Channel selection* (N bits, 1 p/channel)	Switches to select the channel to be configured in the manual and serial operation modes.
	*Amplitude control* (6 bits)	The manual amplitude control comprises six switches, that codify (in binary) the amplitude setting, from 0 (000000) to 48 mA (110000).
	*Mode selector (1 bit)*	Switch to change the operation mode of the PG-nFES: manual or serial
	*Emergency stop (1 bit)*	It stops or restarts the pulse generation of all (middle, lower zone of Figure 1B) when its logic level is set to high or low. When used to restart stimulation, the last configured parameters of each channel are maintained
Serial interface	*Serial protocol*	Channel type: Simplex (only receives data from external devices) DATA transfer speed: 9600 bauds (bits per second) DATA format: 1 start bit, 8 data bits (LSB first), 1 start bit, 1 stop bit, no parity bit. Burst-time: The 3 LSBs codify the 5 possible values (0, 1, 2, 3 and 4 s). Amplitude: The 6 LSBs codify the 48 possible values (1–48 mA. Resolution: 1 mA) Frequency: The 8 bits codify the 150 possible values (1–150 Hz. Resolution: 1 Hz) Pulse-width: The 6 LSBs codify the 50 possible values (10–500 μs. Resolution: 10 μs)
	*Buffering*	Accumulates 4 consecutive serial data packets received (10 bits each), one for each parameter
	*Deserialization*	Upcoming serial data are separated into four bytes, one for each parameter and sent separately sent to the Operation Mode controller (Figure 1B, left side), where parameter settings are stored in dedicated registers for each channel

(*a*) Once an operation mode is selected (*Mode Selector* switch*)*, the system sets the initial values of the stimulation parameters. For the manual mode, burst time: 3 s ON / 7 s OFF, pulse width: 300 μs, frequency: 30 Hz, and amplitude: 0 mA. For the serial mode, burst-time: 0 s OFF (continous stimulation), pulse width: 0 μs, frequency: 0 Hz, and amplitude: 0 mA.

(*b*) The C*hannel Selection Switches* define the active channel (n), whose parameters can be set by the external *Amplitude Switches*, or by serial commands received by the HC-06 bluetooth module (Figure 1B, lower left), connected to the internal *Serial Interface* module inside the FPGA. The configuration and updating of parameters are controlled by the manual amplitude controller or the serial interface module, depending on the operation mode selected.

(*c*) The *Operation Mode Controller* module continually reads its inputs and stores the parameter settings in dedicated memory registers for each channel.

(*d*) Whenever new values are stored in the *Parameter Registers* module, the outputs of the *Operation Mode Controller* module are updated for the active channel, while keeping the last settings in all other channels.

#### Generation of Pulses

Each *CHn* module generates the stimulation pulses and bursts on the n-th channel (Figure 1A). The whole range of values available for each parameter are stored in individual *Look Up Tables* (LUT) for the *burst-time*, *pulse amplitude* and *pulse frequency control* modules (Figure 1A).

##### Burst-Time Control Module

Two intermediate variables, frequency and duty-cycle, are inputs for the *Burst-Time Generator* module that, in combination with a MUX, control the timing of the burst of pulses. Four values are obtained from the *Burst-time Controller module*: two for the *Pulse amplitude control* (frequency and pulse width of the PWM signal) and two for *Pulse Frequency Control* (pulse width and frequency of the monophasic pulses) modules.

##### Pulse Frequency Control Module

This module takes as inputs the outputs of the *Pulse Width* and *Frequency LUTs*, and in turn outputs two variables (one for the pulse width parameter and one for the frequency parameter). These variables are used as inputs to each *Monophasic Pulse Builder* module, where they are used to generate a pair of digital pulses with the value of the pulse width parameter and a 10 μs delay between them (repeated at time intervals defined by the frequency variable).

##### Amplitude Control Module

The control of amplitude is carried out first in the digital domain, involving the FPGA and the DAC’s input, and later in the analog domain, involving the DAC’s output, the mixing circuits and the output stage (Figure 1B, right side). This module takes as inputs two intermediate variables coming out from the burst-time controller module: a frequency and a duty cycle of a PWM signal. These two values are associated to LUTs’ addresses, that contain the value of counters that define frequency (1 kHz) and the duty cycle (0–98%) of a PWM signal, the output of this module (Figure 1A).

The output PWM signal is used as input to a 4-channel, 12-bit resolution DAC (LTC2645), which translates the PWM signal to a DC voltage (0–2.5 V) proportional to the duty cycle. The DC voltage is used in the next stage (signal mixer) to modify the amplitude of the pair of monophasic pulses, so that a 0–96% duty cycle is mapped to 0–48 mA pulse amplitude.

##### Signal Mixer

This block (Figure 1B, right side) integrates the three stimulation parameters in each monophasic pulse. The signal mixer, comprising mainly an NPN transistor (BC546) configured to operate as an ON/OFF switch, transforms the digital pulses (3.3 V) to the amplitude set by the DAC’s output voltage (0–2.5 V) combining the frequency, pulse width and amplitude parameters in each monophasic pulse. Within the signal mixers the signal mixers, the polarity of both pulses is inverted (to normal) before they are sent to the output stage. At the output stage, the two monophasic voltage pulses are combined into a single biphasic, current-controlled pulse, as described in Leyva et al. (2018). A single PG-nFES channel plus the output stage form a complete FES stimulation channel, which underwent technical and application tests as described in the following sections.

### Performance Tests of the Functional Electrical Stimulation System

The performance tests of the FES system were divided in two stages: the technical tests, for verification of the whole range of stimulation parameters settings, and a proof-of-concept, where sequences of electrical stimulation were programmed through the serial interface to produce a set of upper limb movements in healthy participants.

#### Characterization of Stimulation Parameters

The manual operation mode was selected to configure a set of electrical stimulation sequences, for characterization of three parameters: amplitude, frequency and pulse width. For each parameter, the one of interest was varied while the other two remain fixed at some typical value. The parameter settings established for the characterization of each parameter (and the incremental steps in their values) are shown in Table 3. It is worth mentioning that the information in Table 3 is different to that of Table 1, which contains the technical specifications required for the device, including the desired values of stimulation parameters.

**TABLE 3:** Ranges and steps of stimulation parameters used in the characterization measurements.

Fixed parameters values	Measured parameter	Measuring range	Increments
Amplitude: 16 mA Pulse-width: 300 μs	Frequency (Hz)	5–30, 30–150	5, 10
Frequency: 30 Hz Amplitude: 16 mA	Pulse width (μs)	20–50, 50–100, 100–400	10, 50, 100
Frequency: 30 Hz Pulse-width: 300 μs	Amplitude (mA)	1–48	1

The pulses delivered at the output stage were applied to a 1 kΩ load resistor. Five consecutive measurements for each parameter were performed using a current probe (TCP0030A, Tektronix) and a digital oscilloscope (MDO3104, Tektronix) with 1 GHz bandwitdh. For verification and analysis of the data obtained from the measurements, descriptive parametric statistics were calculated for each stimulation parameter: mean, standard deviation and percentage error (shown in Equations 1–3):


(1) $$\bar{X}=\frac{\sum_{i=1}^{N}X_{i}}{N}$$

(2) $$\sigma =\sqrt{\frac{\sum_{i}^{N}{\left(X_{i}-\bar{X}\right)}^{2}}{N}}$$

(3) e=|VExpected-VMeasured|VExpectedx 100%

where $\bar{X}$ is the mean of measurements, σ is the standard deviation, *X_i_* represents each measurement, N is the number of measurements, *e* is the percentage error, *V*_*Expected*_ is the programmed value, and *V*_*Measured*_ is the average of measurements ($\bar{X}$).

Also, for each stimulation parameter the mean absolute percentage error (MAPE), and the coefficient of determination (*r*^2^) were calculated, to assess the overall accuracy and linearity (respectively) of the measurements. Moreover, the repeatability of the characterization measurements for each stimulation parameter was obtained (Portuondo and Portuondo, 2010).

#### Modulation of Stimulation Parameters. Simulation

To test the flexibility of the PG-nFES for the control of stimulation parameters, we implemented three patterns of simultaneous modulation of two parameters with the following characteristics:


(4) $$y1\left(t\right)=\sin\left(2\pi ft\right),f=0.5Hz,t=0.2s,t_{max}=8s$$

(1) Frequency and Pulse Width Modulation:

Frequency range (Hz): 10–90
Pulse Width range (μs): 20–500
Amplitude (mA) = 20 (fixed)

(2) Amplitude and Pulse Width Modulation:

Amplitude range (mA): 1–48
Pulse Width range (μs): 20–500
Frequency (Hz) = 30 (fixed)

(3) Frequency and Amplitude Modulation:

Frequency range (Hz): 20–50
Amplitude range (mA): 1–48 mA
Pulse Width (μs) = 100 (fixed)

The modulation patterns were programmed in MATLAB ^®^ scripts and sent *via* the Bluetooth serial interface of a Laptop PC to the HC-06 receiver connected to the PG-nFES, to update the stimulation parameters as required.

#### Proof-of Concept: Non-invasive Functional Electrical Stimulation

A feasibility test of the potential use of the PG-nFES for transcutaneous FES applications was performed using a single FES channel. For this, the PG-nFES coupled to an output stage (Leyva et al., 2018) through the DAC and signal mixers (right side of Figure 1B), were used to deliver sequences of electrical stimulation pulses to the arm (through transcutaneous stimulation electrodes) of two able-bodied volunteers, with the aim of producing four different movements of the upper extremity. Before the proof-of-concept session, each participant received, read, and signed an informed consent letter, which was approved by the Research and Ethics Committees of the Institution, based on the principles for research in human beings of the Nuremberg Code and the Helsinki Declaration promulgated by the World Medical Association. In that letter, the participants were informed that some photographs and videos would be recorded during the experimental session, that could be used for scientific and educational purposes, without compromising their identity at any moment.

The test consisted in the application of FES sequences, programmed in the PG-nFES and delivered by the output stage (Figure 1), to generate four upper limb movements: hand opening, power grasp, elbow flexion and arm abduction. For each movement, trains of biphasic pulses were applied through a pair of reusable, self-adhesive surface stimulation electrodes (Durastick Plus ^®^, square shaped, 5 cm × 5 cm) placed over the forearm and the upper arm. For the hand opening and power grasp movements the electrodes were placed in the forearm, over the motor points for the wrist and hand extensors and flexors muscles. For these movements, an elastic fabric sleeve was placed over the forearm, with marks in suitable positions of stimulation electrodes for these movements (Martín, 2019). For the upper arm movements, the electrodes were placed over the motor points of the biceps brachii for the elbow flexion, and in the lateral deltoid for arm abduction, as recommended in Baker (2000).

A MATLAB ^®^ script was written to send wirelessly (*via* the Bluetooth serial interface of a Laptop PC) serial messages to the HC-06 receiver, which was hardwired to the Serial Interface block of the PG-nFES (Figure 1, lower-left corner). This way, the corresponding serial commands were sent to the PG-nFES, following the data protocol and format explained in Table 2, to set the desired stimulation parameters. The fixed parameters used in FES sequences for all four movements were 30 Hz frequency, 300 μs pulse-width, and burst-time of 4 s ON, 6 s OFF. For each target movement, the stimulation amplitude was increased from 1 mA, in 1 mA steps, to the level that produced full range of motion without becoming uncomfortable.

#### Modulation of Parameters. Practical Demonstration

**FIGURE 2:**
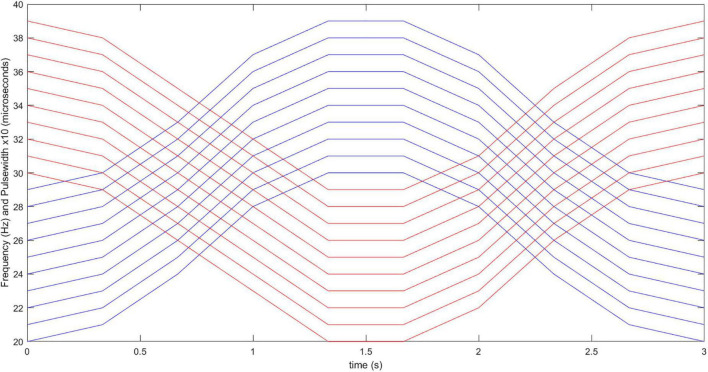
Stimulation pattern with co-modulation of frequency and pulse width. Red and blue lines correspond to frequency and pulse width values throughout the 3-s long stimulation burst, for 10 consecutive repetitions of the functional movements. Initial (0 s) frequency and pulse width values are varied with sinusoidal patterns throughout the time, with updating interval of 0.33 s.

To test the capacity of our system for modulation of parameters in real conditions, we designed a stimulation pattern comprising simultaneous modulation of frequency and pulse width with a sinusoidal profile (Figure 2). Moreover, the frequency (20–40 Hz) and pulse width (200–400 μs) values were chosen within ranges reported as suitable for FES applications while avoiding rapid muscle fatigue (Downey et al., 2011). Moreover, the combinations of frequency and pulse width were selected to maintain almost constant the charge throughout the duration of each individual FES-induced contraction.

To compare the capabilities of the PG-nFES with conventional FES stimulation (with fixed parameters), two stimulation approaches were used to perform the functional movement “bring a bottle to the mouth”: the above-mentioned parameter modulation approach, and the conventional FES approach, where constant frequency (30 Hz) and pulse width (300 μs) are employed. To enable this functional movement, a second complete FES stimulation channel was implemented, taking advantage of the scalability features of the PG-nFES, illustrated in Figure 1B. The stimulation pattern with fixed parameters (30 Hz/300 μs) was used in the stimulation channel used for the power grasp (finger flexors), and the modulation pattern was used in the stimulation channel used for the elbow flexion (biceps brachii).

Electrical stimulation was applied through pairs of squared (5 × 5 cm) electrodes (Durastick Plus ^®^) over the midline of the left biceps brachii muscle and over the motor point of the finger flexors in the left forearm of a healthy volunteer. Functional stimulation thresholds (pulse amplitudes) were determined in two conditions: (1) 25 mA for 30 Hz/300 μs pulses (to be used in the fixed parameters condition), and (2) 22 mA for 40 Hz/400 μs pulses (the highest frequency and pulse width values to be employed in the modulation stimulation pattern). Moreover, the functional threshold for the power grasp movement when using fixed 30 Hz/300 μs pulses was 14 mA. Those stimulation amplitudes were kept constant throughout all the stimulation repetitions for both protocols.

Stimulation sequences for each movement repetition were delivered, starting with Channel 1 (power grasp) presented 0.5 s ahead of Channel 2 (Elbow flexion). The duration of stimulation bursts for Channel 1 was 4 s while for Channel 2 was 3 s, with a 6 s rest time after the stimulation in channel 1 ceased (4 s). This way, there was a 10 s period between the start of each movement repetition. Twenty repetitions of this stimulation sequence were delivered to the participant’s arm, to perform the “bring bottle to mouth” functional movement. A rest time of 20 min was used between the two protocols, and the participant was blinded to the order of delivery.

During the stimulation session, the subject was sitting in a chair with the front side rotated 90° relative to the front view angle of the camera. Green squared markers of approximately 1 cm^2^ were placed in the wrist, elbow, and shoulder joints, to facilitate the extraction of data. In the rest time between stimulation repetitions the bottle of water lied over another chair placed at the side of the participant, while its hand rested over the same chair, with the fingers positioned around the bottle.

##### Video Recording and Analysis

The sequence of 20 movement repetitions for each stimulation condition was recorded on video (c922 Pro, Logitech) at 720 p resolution and 30 frames per second, with the software Logitech Capture (v 2.02.155) running in the same computer used to send the serial commands to the PG-nFES. The video recordings of both stimulation protocols were analyzed with the Kinovea software (v 0.8.15), using the tools to set and follow the markers previously positioned over the bone prominences of the shoulder, elbow and wrist joints, and calculate the angle of the elbow joint throughout the movements. Finally, the minimum elbow joint angle (corresponding to the highest elbow flexion angle) was identified for each movement repetition and protocol. For each protocol, the mean and S.D. of the elbow joint angle measurements were calculated and ploted, and linear regressions were performed.

## Results

### Characterization of Stimulation Parameters

**FIGURE 3:**
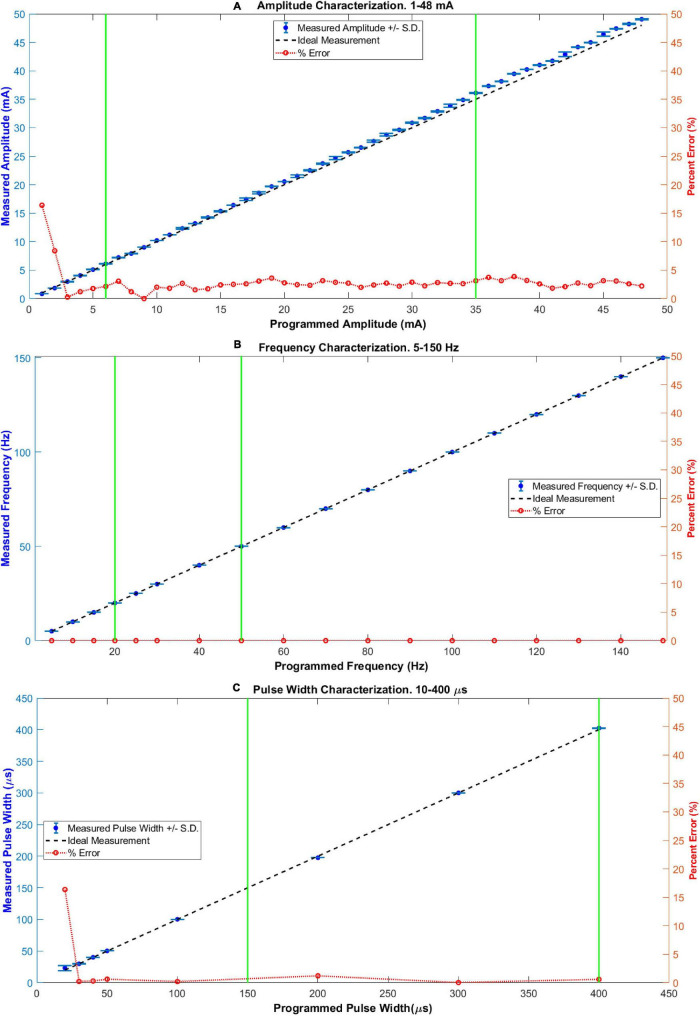
Characterization of stimulation parameters: **(A)** amplitude, **(B)** frequency, and **(C)** pulse width. In each case, the values of parameters among the two vertical green lines define a range commonly used for upper limb FES applications. The maximum percentage error (%) inside these zones was 3.6, 0, and 1.21, for incises **(A–C)**, respectively.

Figure 3 shows the ideal response, the average, standard deviation, and percentage error obtained in the characterization measurements for the three stimulation parameters: amplitude (Figure 3A), frequency (Figure 3B) and pulse width (Figure 3C). From Figure 2, it can be seen that highly linear responses are obtained for the three stimulation parameters with *r*^2^-values of 0.9998 for the amplitude and pulse width and 1.0000 for the frequency. The corresponding MAPE values were 2.84% for the amplitude, 2.44% for the pulse width, and 0% for the frequency parameter (no errors). The repeatability was 87 and 81.5% for the amplitude and pulse width, while for the frequency the formula was not suitable (having a MAPE of 0%). The statistical quantities obtained from the characterization are concentrated in Table 4 for quick reference.

**TABLE 4:** Results of the statistical quantities obtained from the characterization measurements of the stimulation parameters.

	MAPE (%)	*r* ^2^	Repeatability (%)
Amplitude	2.84	0.9998	87
Pulse width	2.44	0.9998	81.5
Frequency	0	1.0	1

Noticeable errors (over 5%) in the measurements were present only for the two smallest amplitude settings (16.4% and 8.4%, for 1 and 2 mA, respectively) and for the smallest pulse width setting (16.36%, for 20 μs). Within the range of parameters commonly used for non-invasive upper limb FES applications (marked in Figure 3 between two green vertical lines for each parameter; 6–35 mA for amplitude, 20–50 Hz for frequency, and 150–400 μs for pulse width), the maximum absolute percentage errors were 3.6, 0, and 1.21%, for the amplitude, frequency, and pulse width, respectively.

**FIGURE 4:**
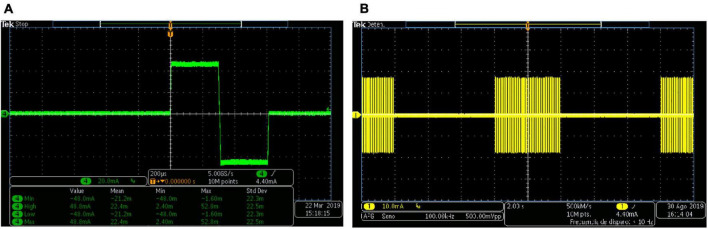
Biphasic stimulation pulses obtained with the PG-nFES. **(A)** A single stimulation pulse, with 48 mA amplitude (96 mA peak to peak) and 200 μs pulse width. **(B)** Bursts of stimulation pulses, with burst-time of 4 s ON, 6 s OFF, and peak amplitude of 16 mA.

Figure 4A shows the capture of a biphasic current-controlled pulse, while Figure 4B shows three consecutive bursts of pulses, as they leave the output stage, measured by the current probe of the oscilloscope through the 1 kΩ load resistor. In Figure 4A, the desired 300 μs pulse width, and 48 mA peak amplitude of the biphasic stimulation pulse, can be observed. In the middle burst of Figure 4B, the burst-time of 4 s ON, 6 s OFF, and the peak pulse amplitude of 16 mA can be appreciated.

### Modulation of Stimulation Parameters. Simulation

**FIGURE 5:**
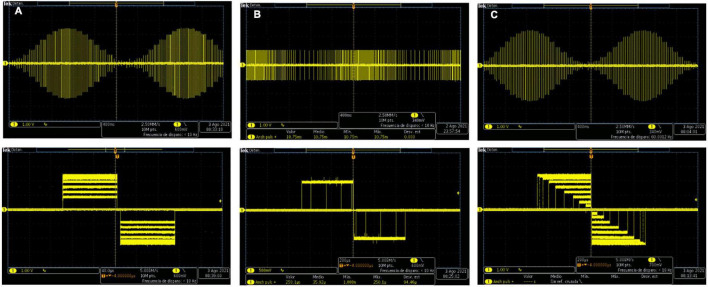
Simultaneous modulation of pairs of two stimulation parameters with the PG-nFES. **(A)** Frequency and Amplitude Modulation, **(B)** Frequency and Pulse Width Modulation, **(C)** Amplitude and Pulse Width Modulation. In the corresponding column for each incise, the upper plot illustrates the slow changing modulation (0.5 Hz) of one parameter (amplitude or frequency), and the bottom plot shows the modulation of the other parameter (amplitude or pulse width).

**FIGURE 6:**
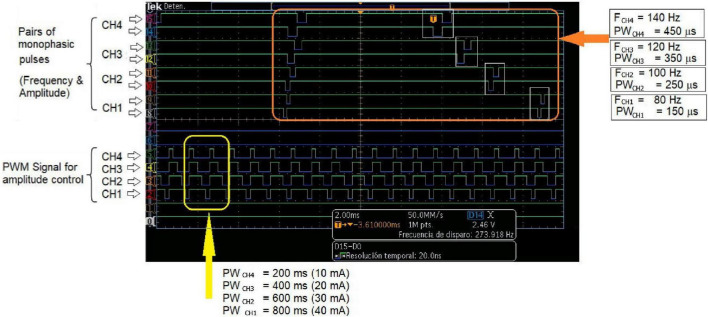
Multichannel operation of the PG-nFEs. Top: Pairs of monophasic pulses corresponding to four stimulation channels. The small white rectangles inside the big orange rectangle highlight the phase delay between each pair of pulses, required for forming the biphasic stimulation pulses in the output stage. Each channel was programmed with different frequency and pulse width settings, shown at the right of the oscilloscope’s screenshot. Bottom: PWM signals for amplitude control of the four stimulation channels. The yellow rectangle in the left highlights the difference in duty cycle of the signals. The bottom text indicates the nominal value of the pulse width and amplitude for each channel and the corresponding settings of stimulation amplitude.

Figure 5 shows three different combinations of simultaneous modulation of two (of the three) stimulation parameters. This illustrates the flexibility of the PG-nFES for setting the stimulation parameters. In the lower plots of Figure 5, the persistence of the oscilloscope was increased to enable the visualization of different values of the modulated parameter within a 1-s time window. On the other hand, Figure 6 shows the digital output signals that the PG-nFES generates for four stimulation channels. For each channel the pairs of monophasic pulses can be appreciated, which contain the frequency and pulse width information, and the PWM signals, which convey the amplitude information for each channel. It is worth noting that all channels had different values for the amplitude, frequency and pulse width parameters, and that the corresponding control signals were active at the same time. Although this was not illustrated in Figure 5, the parameters of any channel can be modified without affecting the other parameters of the same or the other channels.

### Feasibility Test: Non-invasive Functional Electrical Stimulation

**FIGURE 7:**
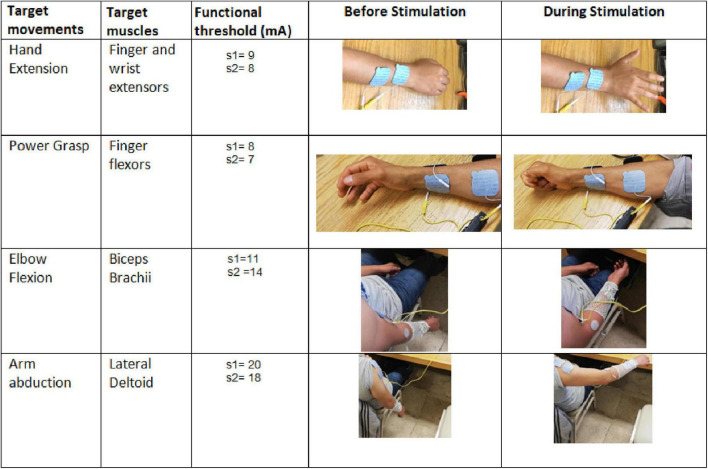
Proof-of-concept of the PG-nFES for non-invasive transcutaneous FES applications. In the first two columns (from the left), the name of the target movements and muscles are shown. The third column shows the functional thresholds used for each subject to generate each target movement. The fourth and fifth columns contain pictures of the hand or arm before and during (respectively) the delivery of the bursts of stimulation pulses to the target muscles.

Figure 7 shows the FES-induced upper limb movements achieved with two healthy volunteers (both males, and 28 years old) by using a single FES stimulation channel. The stimulation parameters and the target muscles over which electrodes were positioned, are presented for the hand opening (Figure 7A), power grasp (Figure 7B), elbow flexion (Figure 7C), and arm abduction (Figure 7D) movements. The four movements were achieved in both subjects (right arm), without any report of discomfort due to the electrodes, the stimulation pulses, or the induced movements.

### Modulation of Parameters. Practical Demonstration

**FIGURE 8:**
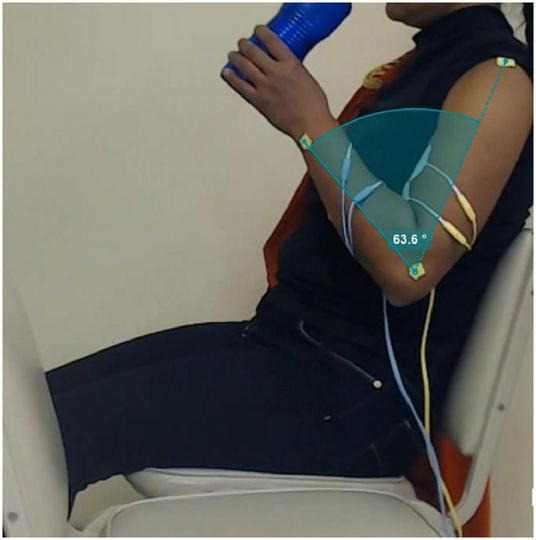
Picture extracted from a frame of video recording of the “bottle to mouth” functional task. The green markers positioned in the shoulder, elbow and wrist joints are used in the Kinovea software to measure the elbow angle for each movement repetition, as illustrated in the green-shaded area.

**FIGURE 9:**
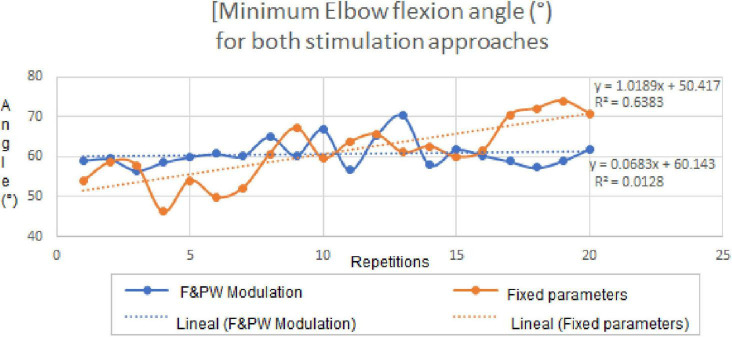
Elbow joint angles for the fixed parameters and co-modulation stimulation protocols throughout the 20 repetitions of the “bottle to mouth” functional task. Linear regression equations and plots for the achieved angles with each stimulation protocol, are shown inside the graph.

The 20 repetitions of the functional movement “bring the bottle to the mouth” were achieved with each of the two stimulation protocols. Figure 8 shows a screenshot of the video recorded during a single repetition of the movement, showing the positions of the joint markers and an example of the calculation of the elbow joint angle in the Kinovea software. In that picture, we can appreciate the experimental setup including the participant sitting in the chair, the grasped bottle near the mouth and the markers in the corresponding joint bone prominences. Figure 9 summarizes the results of this experiment, where the linear regression equations obtained for both protocols are shown, with slopes of 1.01 and 0.06, respectively, for the protocols with fixed parameters and co-modulation of frequency and pulse width. Moreover, the average elbow joint angle for both protocols were very similar, with 60.86° +/− 3.56° for the modulation pattern, and 61.15° +/− 7.54° for the fixed pattern.

## Discussion

In this paper, the functionality of a pulse generator based on an FPGA architecture and its feasibility for non-invasive upper limb FES applications (PG-nFES), were verified through technical characterizations and a proof-of-concept for upper limb FES, when combined with a previously designed output stage (Gutiérrez et al., 2018), that provides biphasic, current-controlled electrical stimulation pulses. By setting the PG-nFES with commonly used stimulation parameters for non-invasive neuromuscular applications and surface stimulation electrodes, upper limb movements of the hand and arm (involving the elbow and shoulder joints) were achieved (Figure 7). In contrast, many FPGA-based stimulation systems reported in the literature are targeted to invasive FES applications (Castelli et al., 2017) or are not aimed for upper limb applications (Chen et al., 2009). On the other hand, recent works reporting FES systems for the upper limb (De Marchis et al., 2016; Kutlu et al., 2016; Annetta et al., 2019; Torah et al., 2019; Ward et al., 2020), are designed for custom arrays of stimulation electrodes aimed to small muscles of the forearm to achieve wrist and finger movements. However, these electrode arrays are only available at research settings, which limit their use in the clinics and the adaptation to other FES applications. In contrast, our system is compatible with standard-sized clinical electrodes for transcutaneous stimulation, which can be placed over a variety of body parts and FES applications.

The current level of development of the PG-nFES, according to NASA’s TRL (Technology Readiness Levels) scale (Mankins, 2009), is TRL 3 (Experimental critical function and characteristic proof-of-concept). Hence, in this work we focused on the technical validation of the system and performed basic proofs-of-concept of non-invasive FES with one and two-channels.

Regarding the characterization of stimulation parameters, it was useful to show the technical feasibility of the system to deliver biphasic stimulation pulses with parameter values within the ranges commonly used in upper limb non-invasive FES applications (Figure 2). This electrical bench test characterization was performed in the required ranges of operation, as needed in an initial proo-of-concept for a technology at the TRL3/TRL4 development level. From the statistical parameters in Table 4, we can see that the system is highly accurate and repeatable in the frequency parameter, and slightly less accurate in the amplitude and pulse width. Regarding linearity, high values were obtained for the three parameters, with an *r*^2^ coefficient of 0.9998 for both the amplitude and the pulse width, and 1.0 for the frequency (since there were no errors in the measurements). Together, these results support the technical feasibility of the PG-nFES.

The amplitude errors at small values have two possible causes: the minimum measurable value specification of the current probe (1 mA), or a slight DC offset introduced by the output stage (or a combination of both). Regarding the frequency parameter, the expected value was obtained by the oscilloscope’s algorithm for each individual measurement (0% percentage error). For future works, the potential sources of errors will be assessed before adjusting the design and repeating the characterization procedure.

Currently, the maximum selectable amplitude in the PG-nFES is 48 mA peak, which is enough for most non-invasive FES rehabilitation applications for the upper limb. However, the output stage which was combined with the PG-nFES can deliver biphasic, current-controlled pulses over 80 mA of peak amplitude (Leyva et al., 2018). In the future, an updated version of the output stage will be implemented for a multichannel FES system. Moreover, the use of a 12-bit resolution DAC enables future refinements in the amplitude resolution (0.5 mA or less), which has been considered as relevant to improve upper limb FES applications (Schauer, 2017). Furthermore, being the LTC2645 a four-channel DAC based on PWM input signals (without serial interface), the scaling procedure to multichannel systems can be greatly simplified, as illustrated in Figure 6.

When compared to works with similar aims, the FES system based on the PG-nFES can achieve current stimulation pulses of high amplitude (48 mA, suitable for conventional electrodes available at clinics) while allowing total flexibility in the selection for each stimulation parameter and for each stimulation channel. In contrast, other FES systems limit the amplitude to low values (Annetta et al., 2019) or to be the same in all channels (Ward et al., 2020). Regarding the pulse frequency in other FES systems it depends on the number of active channels (Annetta et al., 2019) or is fixed for all of them (Ward et al., 2020). In contrast, our system allows frequencies from 1 to 150 Hz for each channel and amplitudes from 1 to 48 mA, as shown in Figure 3. Also, multiple channels can be operated and active at the same time, while keeping that flexibility in the selection of stimulation parameters, as shown in Figure 6, where different stimulation amplitudes, frequencies and pulse widths were used for each of four active channels.

In the proof-of-concept the PG-nFES was used to produce movements of different segments of the upper limb (hand, forearm, shoulder). Moreover, a second FES stimulation channel was implemented, taking advantage of the scalability feature of the PG-nFES architecture, while maintaining the flexibility in the configuration of stimulation parameters. To prove further the capabilities of the PG-nFES, the two FES channels were used to perform a clinically relevant functional movement related to a key activity of daily living: “bringing a bottle of water to the mouth.” For this, overlapping stimulation sequences for the hand finger flexors and the biceps brachii muscles were required. Different types of stimulation parameters were used for each channel: fixed parameters in one channel (finger flexors) and a pattern of co-modulation of frequency and pulse width in the other channel (biceps). This demonstrated that the scalability and the flexibility of the PG-nFES, since no adjustments to the FPGA firmware were needed.

In the experiment with two stimulation channels, a single participant was included, since the purpose was to show the scalability feature and the flexibility in the configuration of the stimulation parameters. For future developments of the system (TRL 5/TRL 6), further tests will be performed with upper limb movements of the upper limb in unimpaired and impaired subjects (validation in relevant environment). This will be a step forward toward validating the potential of the system for clinical purposes. Also, a complete study of the repeatability of FES-induced movements with the PG-nFES can be performed, comprising several repetitions of different upper limb movements and measurement of electrophysiological and kinematic variables (i.e., sEMG, goniometry, orientation, or acceleration of the limbs). A plausible hypothesis is that, once the stimulation parameters were calibrated for each subject, similar values (before muscle fatigue arises) will be found in those variables across repetitions and/or individuals. Other strategies can be implemented to limit or delay the effects of muscle fatigue, and improve the performance in FES applications, like the pattern of modulation of parameters proposed here and others reported in the literature (Ibitoye et al., 2016).

The complexity of other FPGA-based systems (Annetta et al., 2019) requires that the device in charge of controlling the FES application (PC or embedded) updates constantly the value of each parameter for each single pulse. In our proposed FPGA-based architecture this is not necessary, since the last parameter settings are stored and maintained fixed unless a new parameter update is requested by the application. Also, a pulse generation burst-mode is considered in the PG-nFES, where the active and rest time are chosen as another stimulation parameter, with constant burst stimulation as a special case. Using this feature of the PG-nFES a high flexibility is possible in the parameter settings, since any of them can be updated while the burst-time setting is maintained or adjusted, as required by the application. Examples of the flexible features of the PG-nFES are shown in Figures 5, 6, and especially in Figure 2, where the pattern of co-modulation of frequency and pulse width, for 10 consecutive movement repetitions, is illustrated. Those features were used in the two-channel stimulation sequences for the power grasp and elbow flexion movements, where the burst-mode was used for the power grasp channel (with fixed frequency and pulse width, and amplitude adjusted to the user’s personal threshold), while for the elbow flexion channel the continuous stimulation mode with co-modulation of frequency and pulse width was employed. It is worth noting that all the configuration and update of the stimulation parameters were performed wirelessly, sending the required commands (Table 2) from MATLAB scripts to the FPGA, *via* a Bluetooth serial interface. This is relevant for safety and portability, since no physical connection to a controlling device is required.

Currently, two of the six available outputs of the PG-nFES have been connected to corresponding DAC channels, signal mixers (amplitude control) and output stages (Figure 1B). Together, they complete two FES stimulation channels. However, within the FPGA all the digital modules for six stimulation channels (Figure 1B, left side) were built and functioning. For each CHn module, the system can generate simultaneously the three digital signals required: 2 monophasic pulses for frequency and pulse width + 1 PWM signal for amplitude control, which were illustrated in Figures 1A, 6. In Figure 6, the scalability feature of the system is illustrated for the case of four channels. The architecture of the PG-nFES was helpful to simplify the scaling of the system to multiple channels, through replication of digital modules inside the FPGA and external analog circuits (Figure 1B). With the implementation of the functional task movement involving two simultaneous movements, each one with different stimulation patterns (fixed and modulated), the potential for a multi-channel version of the system was shown. Such system implementation would allow the use of independent closed-loop controllers for different channels and parameters.

Regarding the modulation pattern, it is relevant to mention that it was designed combining ideas from previous works related to co-modulation of frequency and pulse width for fatigue reduction (Gregory et al., 2007; Chou et al., 2008; Downey et al., 2011), and others suggesting that stimulation frequency should be continually increased during consecutive movement repetitions (Chou et al., 2008). Also, other works were considered that proposed the use of different combinations of pulse width and frequency with equivalent total charge, since they induced constant torque levels (Gregory et al., 2007). The results of the feasibility test of the co-modulation pattern applied to a functional movement, agree with the literature reporting delayed muscle fatigue and improving the performance in FES applications. Future versions of the system would make use of the frequency and pulse width co-modulation approach in patients with impaired CNS conditions that present spasticity, such as spinal cord injury. Using a gradual, variation of the two parameters (Figure 2), while using constant pulse amplitude, can help to reduce the spastic reflex when applying stimulation, by using parameters suggested in the literature for that end (Hui-Chan and Levin, 1993; Bekhet et al., 2019). This is of main importance since spasticity, as measured by the Ashworth scale, is generally used as an exclusion criterion in clinical FES protocols.

Regarding the results of the functional movement task (Figure 9), the linear regression equations for the elbow joint angle throughout the repetitions (time), have a much lower slope for the fixed parameter pattern. This may indicate a higher stability in the achieved functional movements with the parameter modulation pattern, even when frequency and pulse width were varied during the 3 s-long burst of pulses. Furthermore, the results suggest that the modulation pattern of frequency and pulse width was more effective to maintain the elbow angle more stable (half the S.D) than the fixed pattern, throughout the 20 repetitions. It is worth mentioning that, during the stimulation tests using the modulation protocol the participant reported the biceps contraction felt “stronger toward the end”, and the subject was blinded to what type of protocol was being used at any time. This can be further investigated in future works where sEMG electrodes and inertial sensors were used to assess muscle activation and limb movement during the whole experiment. This additional bioelectric and kinematic information can help us to better understand this effects of the stimulation pattern and others.

In the future, the proposed FPGA-based architecture of the PG-nFES could be used to develop a flexible multichannel stimulation system, involving multiple target structures being stimulated in a coordinated manner, as required for non-invasive upper limb FES therapy (Kapadia et al., 2020). When combining such multichannel FES system with a properly designed rehabilitation intervention and clinical study, the potential of this sytem for motor neurorehabilitation could be assessed thoroughly. For this, the technology must be risen from the current TRL3 (proo-of-concept) to at least TRL 6 (prototype tested in relevant environment), according to NASA’s classification (Mankins, 2009).

The FPGA-based PG-nFES architecture was designed to allow independent control of stimulation parameters, as shown in the examples of modulation of parameters in Figure 5. Furthermore, this feature was used to implement a stimulation pattern with co-modulation of frequency and pulse width, which was used to deliver stimulation pulses through one stimulation channel to the biceps muscle (elbow flexion), while using a typical FES pattern with fixed frequency and pulse width, in a second channel, for the finger flexors (power grasp). Combining both movements, several repetitions of a functional task could be implemented, as shown in Figures 8, 9. This way we proved the versatility and flexibility of our system.

In the future, the PG-nFES could be used to implement other parameter modulation patterns reported to reduce FES-induced muscle fatigue (Zhou et al., 2016), such as amplitude-modulated pulse-width modulation Furthermore, the system’s flexibility in handling the stimulation parameters, can be exploited to implement FES applications based on spatially and time distributed stimulation strategies, which promise to reduce muscle fatigue (Nguyen et al., 2011; Maneski et al., 2013) and increase force (Buckmire et al., 2018).

We identify two main contributions of the PG-nFES to the design of FES systems, to mention:

(1) The architecture of the PG-nFES (Figure 1) enables easy scaling to multiple stimulation channels, since additional logic blocks can be configured in the FPGA to replicate the control logic for the extra channels. This was illustrated schematically in Figures 1, and in Figure 6 with digital signals for the control of 4 channels at the output of the PG-nFEs. Finally, the scalability feature it was demonstrated with the implementation of two complete FES channels, and their application in a functional task involving finger and elbow movements (Figure 8).
(2) The PG-nFEs allows high flexibility in the selection of stimulation parameters, even in the multichannel case. Again, this feature was illustrated in Figures 1A, 6, and demonstrated in practice with the functional task “bringing the bottle of water to the mouth,” where two different stimulation patterns were used for each stimulation channel: fixed and modulated frequency and pulse width.

These features are possible, in part, by using Lookup Tables with all the timer values required to handle the four stimulation parameters (burst-time, amplitude, frequency, and pulse width) in their whole operational ranges, and individual registers for each stimulation parameter and channel (Figure 1A). Moreover, a simple PWM module inside the FPGA is implemented to control the pulse amplitude for each stimulation channel (combined with the DAC with PWM inputs and the “signal mixer” blocks). In contrast, most electrical stimulation designs (either based on microcontrollers or FPGAs) use serially controlled (SPI or I2C) DACs to build the pulse shape, width, and amplitude, completely within the DAC (Nogueira et al., 2017). However, it is simpler and more efficient to implement one PWM module inside the FPGA for each stimulation channel, than to implement an SPI communication module for handling as many DACs as stimulation channels.

It is important to emphasize that, besides the technical features of the system mentioned above, biphasic rectangular shaped stimulation pulses, with constant current, are used, which are preferred for safe and efficient non-invasive FES stimulation. In contrast, other FPGA-based designs simplify the pulse shape (Annetta et al., 2019), or keep fixed the value of frequency or amplitude parameters in all channels (Ward et al., 2020), to enable multichannel operation.

Finally, the capabilities of the PG-nFES enable the development of highly customized electrical stimulation sequences for a variety of users and applications. Hence, this architecture can be used as base for the design of robust devices for FES and other electrical stimulation modalities where flexible selection of stimulation parameters in multiple channels might be relevant. Such is the case of brain or spinal cord stimulation, for neuromodulation and rehabilitation applications. In our case, the next step is to integrate the PG-nFES as a component of a multichannel FES-based neuroprosthesis, controlled by a Brain-Computer Interface based on evoked potentials, which can be used for upper limb motor rehabilitation of stroke and spinal cord injury patients.

## Data Availability Statement

The datasets presented in this article are not readily available due to Institutional Policies. Requests to access the datasets should be directed to the corresponding author.

## Author Contributions

JG-M: project conception and evaluation of the proof of concept, writing, review, and acceptance of the manuscript. JM-G: design and implementation of the pulse generator, FES channels, characterizations and proof-of-concept, data acquisition and analysis, writing, review, and acceptance of the manuscript. RD: implementation and experimental tests of the pulse generator, review, and acceptance of the manuscript. IH-P: output stage design and tests with the pulse generator, review, and acceptance of the manuscript. JQ-F, AV-H, and LL-S: data interpretation, writing, review, and acceptance of the manuscript. All authors contributed to the article and approved the submitted version.

## Conflict of Interest

The authors declare that the research was conducted in the absence of any commercial or financial relationships that could be construed as a potential conflict of interest.

## Publisher’s Note

All claims expressed in this article are solely those of the authors and do not necessarily represent those of their affiliated organizations, or those of the publisher, the editors and the reviewers. Any product that may be evaluated in this article, or claim that may be made by its manufacturer, is not guaranteed or endorsed by the publisher.
